# Differential Experiences of Mental Health among Transgender and Gender-Diverse Youth in Colorado

**DOI:** 10.3390/bs11040048

**Published:** 2021-04-09

**Authors:** Brittanie Atteberry-Ash, Shanna K. Kattari, Vern Harner, Dana M. Prince, Anthony P. Verdino, Leonardo Kattari, In Young Park

**Affiliations:** 1 School of Social Work, University of Texas Arlington, 211 S Cooper St., Arlington, TX 76019, USA; 2 School of Social Work and College of Literature, Science, and the Arts Department of Women’s and Gender Studies, University of Michigan, Ann Arbor, MI 48109, USA; skattari@umich.edu; 3 School of Social Work, University of Washington, Seattle, WA 98105, USA; vharner@uw.edu; 4 The Jack, Joseph and Morton Mandel School of Applied Social Sciences, Case Western Reserve University, Cleveland, OH 44106, USA; dmp137@case.edu; 5 School of Social Service Administration, University of Chicago, Chicago, IL 60637, USA; anthonypverdino@gmail.com; 6 School of Social Work, Michigan State University, East Lansing, MI 48824, USA; kattaril@msu.edu; 7 Graduate School of Social Work, University of Denver, Denver, CO 80208, USA; InYoung.Park@du.edu

**Keywords:** depression, suicidality, transgender, mental health, gender diverse, nonbinary, gender identity, youth, NSSI

## Abstract

Young people experience a variety of mental health concerns, including depression, non-suicidal self-injury, and suicidal ideation. These issues are at even higher rates among transgender and gender-diverse (TGD) young people, due to the additional burden of having to navigate a world in which transphobia impacts them at the individual, organizational, and policy levels. However, much of the extant research focuses only on comparing TGD youth to cisgender counterparts. This study explores the nuance within the TDG youth population regarding mental health, examining how gender, race/ethnicity, and sexual orientation change the likelihood of experiencing each of these mental health concerns. Among a sample of over 400 young people, findings indicate that those TGD young people who do not identify themselves within the masculine/feminine binary and those with marginalized sexual orientations were two to three times more likely to experience adverse mental health outcomes, as compared to their peers who are questioning their gender, and who are heterosexual. The implications for mental health professionals and others who work with young people are to recognize that mental health is not a one-size-fits all model for young TGD people, and that the intersection of multiple marginalized identities, must be addressed in order to improve the mental health of this group of young people. Findings can also be used to better understand issues of stigma, discrimination, and victimization in education, health care, and beyond.

## 1. Introduction

Young people in society tend to experience a variety of concerns regarding their mental health, including depression, non-suicidal self-injury (NSSI), and suicidal ideation. These issues are seen at even more elevated rates among transgender and gender-diverse (TGD) young people, given the additional burden of having to live within this world, in which transphobia and cisgenderism impact TGD individuals at the interpersonal (micro), organizational (mezzo), and societal/policy (macro) levels. However, the most existing research tends to focus only on viewing TGD youth as a single gender, comparing them to their cisgender peers. This study aims to explore some of the nuance within the TDG population of young people regarding mental health, looking at how gender, race/ethnicity, and sexual orientation intersect to affect the likelihood of experiencing depression, NSSI, and suicidal ideation.

### 1.1. Transgender and Gender-Diverse Identities

Extant research suggests that approximately 0.6% of adults in the United States are somewhere on the transgender spectrum, but this only includes transgender identity and does not include nonbinary identity [1]. However, young people (specifically those under the age of 24) are more likely to have a transgender or gender-diverse (TGD) identity than their adult counterparts. In fact, 0.7–3.2% of those in the youth/young adult category identify as TGD [1,2,3]. “Transgender” and “nonbinary” are both broader terms that include other identities within them. These terms are understood to indicate a disconnect or lack of perfect fit between the societal expectations associated with a person’s sex designated at birth and an individuals’ sense of self. This includes both trans men and trans women, as well as individuals whose experiences of gender do not fit within binary and dichotomous models of gender (i.e., man vs. woman). Individuals with this experience may use identity labels of nonbinary, genderfluid, genderqueer, agender, and many other labels. Black, Indigenous, and people of color (BIPOC) [4] who have culturally specific identities (i.e., fa’afafine, māhū, two-spirit) may or may not relate to the language of “transgender” or “cisgender”, and are often unable to accurately note their gender on surveys. Due to the highly personal and subjective nature of experiences of gender paired with inconsistent data collection practices, the numbers of TGD individuals tend to vary between and across sources and studies. As these communities continue to gain access to one another and develop affirming language, and as broader societal environments regarding non-cisgender experiences of gender shift, those with culturally specific identities and those who are transgender and/or nonbinary continue to expand and update the language, terms, and labels used. We, the authors, acknowledge that the acronym of transgender and gender diverse (TGD) might not resonate with each individual in these communities and this population: language remains an imperfect way to attempt to capture the complex human experience of gender. However, we will use the acronym throughout this article in order to be as inclusive, and linguistically efficient, as we can.

### 1.2. Mental Health Among Youth

#### 1.2.1. Depression

The Substance Abuse and Mental Health Services Administration reports approximately 13% of youth aged 12–17 have experienced a Major Depressive Episode [5], including major depressive disorder, persistent depressive disorder, postpartum depression, or seasonal affective disorder. Depression remains one of the leading causes of morbidity among adolescents [6]. Given that there are over 42 million adolescents living in the US [7], the high prevalence of depression among youth cannot be understated and some studies have found higher prevalence of depression among cis-females (compared to cis-males) [8,9].

A focal area is considering possible disparities in depression across racial and ethnic groups. For example, a secondary analysis of the National Survey of Children’s Health (*N* = 30,605) found that for White, Black, and Latinx young people, depression was significantly associated with age, with older adolescents (15–17) demonstrating higher rates of depressive symptoms than other adolescent age groups [10]. Another study (*N* = 594) reported that African American young people have more depressive symptoms than White young people [11].

#### 1.2.2. Non-Suicidal Self-Injury

In their analysis of datasets spanning 597,548 young people from 41 countries the researcher found that nearly 17% of respondents had a history of non-suicidal self-injury (NSSI) and both suicidal ideation and attempts are associated with NSSI such as cutting, burning, and scraping [12]. Young women, especially those who are White and heterosexual, are more likely to self-harm [12,13,14,15,16,17,18]. While NSSI is associated with suicide attempts for women [19], men with NSSI are at even higher risk for suicide attempts [20]. Some studies indicate that young people with NSSI who have attempted suicide are more likely to have a substance use disorder than those with only NSSI and those with NSSI and suicidal ideation [18]. Moreover, NSSI and suicidality have higher rates of comorbid mental health problems like depression and anxiety [18].

The most common reason for engaging in self-harm seems to be relief from thoughts or feelings [12,17]. However, for those who also attempt suicide, dissociation, combating apathy, coping with self-hatred, and avoiding more severe forms of self-injury are more strongly associated with NSSI [17]. Much literature focuses on the prevalent relationship between NSSI and suicidality [15,17,21]. Typically, suicidal ideation first occurs with NSSI thoughts, followed by 1–2 months and engagement in NSSI by about 6–8 months and 1–2 years before suicide attempts [21], which are shown to have a direct connection [15]. Young people with NSSI who attempt suicide endorse NSSI methods that are more severe than those with only NSSI or NSSI and suicidal ideation [18] and show higher frequencies and durations of NSSI [14]. Lastly, the presence [16] and severity [18] of depressive symptoms, as well as fearlessness around death [15,16], have been found to facilitate the association between NSSI and suicidality.

#### 1.2.3. Suicidal Ideation

One of the most urgent symptoms of Major Depression as listed in the Diagnostic and Statistical Manual of Mental Disorders 5 (DSM-5) is suicidal ideation (thoughts about one’s death, dying, or killing oneself). The Centers for Disease Control (CDC) report that suicide is the second leading cause of death in the U.S. for young people ages 10–14 and 15–19 [22]. According to The Trevor Project [23], young people experiencing suicidal ideation may engage in suicidal behaviors (e.g., formulating a plan, giving away belongings, saying goodbye, etc.). For high school students, 17% report thinking about attempting suicide in the last year and over 13% made a plan for attempting suicide [24]. Suicidal ideation is associated with differences in gender and is prevalent across school settings (public, private), community levels (e.g., urban, rural, reservation), and race/ethnicity [25,26,27,28,29].

#### 1.2.4. Suicide Attempts

While 17% of high school students reported suicidal ideation and 13% reported making a plan, over 7% had engaged in a suicide attempt within the last year [24]. The fastest growth rate for attempting suicide is in the 12–14 age range [30]. Of note, McKean et al. [31] found that 3/4 of completed suicides occur at the first medically reported attempt with over one-third lacking any prior psychiatric history. When transitioning from suicidal ideation to engaging in a first suicide attempt, young people tend to be triggered by significant interpersonal stressors like a break-up or problems with parents and family [32] and are more likely to report suicidal ideation in the past week, higher severity of lifetime ideation, and a history of suicidal behaviors [19]. Like suicidal ideation, sadness and hopelessness is shown to be associated with a greater likelihood of making a suicide attempt [19,25]. Carbone et al. [30] discovered a seasonal trend for suicidal behavior in that young people are more likely to experience ideation and attempt suicide during the school year, which may be explained by some of the factors described herein.

Suicidality encompasses ideation and behaviors that are related to putting suicide plans into action and suicide attempts/completion. Research has found gender-based differences in suicidality in that cis-males are more likely to die from suicide as a result of the lethality of method used [31,33], but cis-females are more likely to report ideation and attempts (14,30,32). Additionally, White young people are more likely to both attempt and complete suicide (14,32).

Young people who attempt suicide (not completed) are more likely to be diagnosed with a psychiatric disorder [34], but less likely to report past medication use [19]. Those with past ideation and attempts, as well as young people of color, are least likely to use mental health treatment. Lastly, young people who attempt suicide are more likely to report having been exposed to other people’s self-injurious behaviors [34].

### 1.3. Mental Health among Transgender and Gender-Diverse Youth

Nationally, TGD youth are at a significantly increased risk of psychological disorders including major depressive disorder and suicidal behaviors (e.g., ideation, planning, aborted attempt, and attempt) compared to their cisgender peers [35,36,37,38,39]. Community-based samples inclusive of, or based solely upon TGD youth, find rates of major depressive disorder 15%–33; lifetime suicidal ideation 45–95.5%; and lifetime suicide attempt 26–56% [36,38,40,41].

#### 1.3.1. Depression

Research on depression symptoms and major depressive disorder indicates that TGD youth are at heightened risk of both. Using longitudinal data from a community-based study of LGBT young adults in Chicago, Mustanksi and colleagues [36] applied latent class analysis and found that LGBT individuals who experienced either steadily high or increasing rates of victimization from ages 18 to 22 were at higher risk for clinical depression and post-traumatic stress disorder, as assessed by the Diagnostic Interview Schedule for Children, version IV (C-DISC-IV), compared to SGM-young adults who experienced lower or declining rates of victimization [36]. However, it is important to note that only 7.3% of the sample identified as transgender at baseline; and significant attrition was evidenced across waves due to transgender identity. Therefore, it is unclear how TGD youths’ profiles of victimization may differ from their cis-gender peers who are sexual minorities (i.e., lesbian, gay, bisexual or queer). More recently, Guz and colleagues [38] took an intersectional approach to examine rates of depression at the cross-section of sexual orientation and gender identity. Using data from a statewide representative sample of high school students, ages 14–18 years, they found that, compared to cisgender heterosexual youth, transgender heterosexual youth were two times more likely to positively endorse the question, “In the past 12 months, did you ever feel so sad or hopeless almost every day for two weeks or more in a row that you stopped doing some usual activities?” Transgender lesbian, gay or bisexual youths were more than six times as likely to positively endorse this question, and transgender youth who were questioning their sexual orientation were four times more likely than their cisgender heterosexual peers to endorse this question. In a national cross-sectional survey of over 25,000 LGBTQ young people researchers found that TGD young people were more than two times as likely to experience recent depressive mood compared to their cisgender LGBQ peers [39]. Finally, within a community-based nonclinical sample (*n* = 94), 82% of respondents reported current depressive symptoms [42]. Notably, there is wide variation in measurement of depression in these studies; not all current research administers assessment in ways that can be interpreted as clinical diagnosis or of clinical significance.

#### 1.3.2. Non-Suicidal Self-Injury

Systematic reviews of NSSI show higher rates among transgender individuals compared to cisgender counterparts across the life course; prevalence rates of NSSI tend to be higher among younger age groups, and transmasculine individuals appear to report higher levels of NSSI compared to transfeminine [43,44]. One study of transgender youth (*N* = 268) seeking medical treatment at a national gender clinic, lifetime prevalence rate of NSSI was 46.3% and past few months NSSI was reported in 28.7% of youth (44). AFAB patients had significantly higher levels of lifetime NSSI. Higher levels of severe mental illness were also associated with increased rates of NSSI. Transphobic experiences contributed to predicting severity in mental health problems, again highlighting the role of TGD victimization on mental health. Other gender clinic-based samples of adolescents have found self-harming behaviors to be common (between 24–39%) and higher among AFAB individuals [45,46].

#### 1.3.3. Suicidal Behaviors

Studies of TGD youth demonstrate increased suicidality (ideation, planning, attempt). For example, a population-based sample of high school students in California found prevalence rates of past-year suicidal ideation to be almost twice as high for transgender compared to cisgender youth (33.7% vs. 18.9%) [47]. Depression and LGBTQ-based victimization were both significantly associated with increased odds of suicidal ideation. Another population-based state sample tested the influence of TDG identity and sexual orientation on suicidality. In this sample, all transgender youth regardless of sexual orientation were between eight and nine times more likely than their cisgender heterosexual peers to report a past year suicide attempt [38]. A cross-sectional study comparing cisgender LGBQ young people to TGD youth found that TGD youth were over two times more likely to both seriously consider suicide and to attempt suicide when adjusting for age, family income, and race/ethnicity. These odds were slightly less when adjusting for perceived discrimination, or threats due to sexual orientation or gender identity [39]. Lastly, a large (*N* = 896) cross-sectional study of TGD young people found that, at the bivariate level there was a weak association between gender identity and both suicide ideation and suicide attempt. However, when put into the final regression controlling for other variables, transgender identity was not a significant predictor of suicide ideation or suicide attempt, while identifying as male was a negative predictor of suicide attempt [41].

## 2. Materials and Methods

### 2.1. Research Question

In order to add to the literature on TGD youth and the differential experiences of mental health, we pose the questions: (1) How do TDG youths’ experiences of mental health outcomes vary across gender identity?; and (2) How do other identities such as race, age, and sexual orientation affect the likelihood of TGD youth having these experiences?

### 2.2. Methods and Procedures

Data for the current study come from the 2015 Healthy Kids Colorado Survey (HKCS), a statewide survey disseminated to public middle and high schools in Colorado. Data from 2015 were used given that the 2017 and 2019 surveys did not include questions that explored transgender status beyond yes/no, while the 2015 survey includes an item that categorized non-cisgender identities into four groups. The survey sample is selected based on statewide public-school enrollment, then districts, schools, and classrooms are chosen based on random selection. Both the randomly selected district superintendents and principals may opt out of participating in the survey. Additionally, districts and schools not selected for the state sample can opt-in to participation. Further, certain survey questions can be opted out of by request of the district superintendent or a building principal. All schools participating in the HKCS notify parents and guardians that the survey is taking place and that participation is completely voluntary. Districts choose whether to use an opt-in or opt-out policy for parental consent with majority choosing an opt-out approach. In order to best represent enrollment in Colorado public schools, these data are weighted to account for sampling design, school and student nonparticipation and nonresponse, and school population demographics [48].

The 2015 HKCS included a total of 28,151 high school students within two modules of the survey. Questions related to our dependent variables only appeared on Module B; therefore, 14,071 records were automatically dropped. An additional 344 records were dropped due to participants selecting they did not understand the question inquiring about transgender identity. Given that this study examines outcomes only for students who identified under the transgender umbrella, all cisgender respondents were dropped (*N* = 12,543), as well as all missing cases for gender identity (*N* = 633). Next, records missing cases for the dependent variables were dropped (recent depression, *N* = 24; NSSI, *N* = 9; suicide attempt, *N* = 81). Lastly, participants who reported their age was under 14 were dropped (*N* = 30) given that these ages are outside the standard age range for students in high school in Colorado. The previous steps brought the analytic sample to 416. Next, missingness was examined.

In examining the independent variables, missing data ranged from a low of 0% for age and gender identity to a high of 3.6% for sexual orientation. No variables were missing at more than 5%. Given this, listwise deletion was used, which brought the final analytic sample to 396 [49].

### 2.3. Measures

The dependent variable of experiencing past-year depression was captured with the question, “During the past 12 months, did you ever feel so sad or hopeless almost every day for two weeks or more in a row that you stopped doing some usual activities?” with a yes/no response set. Those who indicated yes were coded as 1 with all others coded as 0. To capture the dependent variable regarding NSSI, respondents were asked, “During the past 12 months, how many times did someone you were dating or going out with physically hurt you on purpose?” (Count such things as being hit, slammed into something, or injured with an object or weapon), with a response set of 0 times, 1 time, 2 or 3 times, 4 or more times. This variable was recoded into a dichotomous yes/no variable. The last dependent variable regarding suicide attempts was captured with the question, “During the past 12 months, how many times did you actually attempt suicide?” with a response set of 0 times, 1 time, 2 or 3 times, 4 or 5 times, 6 or more times. This variable was recoded into a dichotomous yes/no variable.

Age was collected with the question, “How old are you?” The response set included the following options: 12 years old or younger, 13 years old, 14 years old, 15 years old, 16 years old, 17 years old, or 18 years old or older (recoded to 18). In order to collect information on race/ethnicity respondents were asked two questions, “What is your race (select one or more)?” Options included American Indian or Alaska Native, Asian, Black or African American, Native Hawaiian or Other Pacific Islander, or White. The next question was, “Are you Hispanic or Latino?” with a yes/no response set. These two questions were combined to create a new variable which included a category for multiracial respondents. For sexual orientation, respondents answered the question, “Which of the following best describes you?” and were given a response set of Heterosexual (straight), gay or lesbian, bisexual, and not sure. For gender identity, participants were asked, “A transgender person is someone whose biological sex at birth does not match the way they think or feel about themselves. Are you transgender?” with responses of No, I am not transgender; Yes, I am transgender and I think of myself as really a boy or man; Yes, I am transgender and I think of myself as really a girl or woman; Yes, I am transgender and I think of myself in some other way; I do not know if I am transgender; and, I do not know what this question is asking.

### 2.4. Data Analysis

All data were analyzed using Stata 16.1 (Stata, 2019). We ran descriptive statistics by mental health challenge indicators (recent depression, NSSI, and suicidal attempts, respectively), followed by three logistic regression models predicting these three indicators.

## 3. Results

### 3.1. Descriptive Characteristics of Sample

Descriptive statistics are reported in (see Table 1). Over half of youth reporting experienced depression in the last year (*n* = 217, 54.80%), just over half (*n* = 200, 50.51%) of students engaged in NSSI, and 28% (*n* = 112) attempted suicide during the last year.

#### 3.1.1. Depression

Recent depression was highest among youth who identified as White (48.4%), Multiracial (27.2%), followed by Latino/Hispanic (17.5%), American Indian/Alaska Native/Native Hawaiian/Pacific Islander (3.2%), Asian (2.3%), with Black students having the lowest incidence of recent depression in past year (1.4%). Regarding sexual orientation, recent depression prevalence was 39.6% among bisexual students, followed by those who stated they were not sure of their sexual orientation (23.5%), heterosexual (18.9%), with students who identified as gay or lesbian having the lowest rates (18%). For gender identity, students who stated they did not know if they were transgender had the highest rates of recent depression (37.8%). The prevalence of recent depression was 22.6% for transmasculine students and students who identified as transgender and outside the binary of man/woman. Transfeminine students showed the lowest rates of experiencing recent depression (17.1%).

#### 3.1.2. NSSI

The rates of NSSI in the last year was highest among youth who identified as White (49.0%), Multiracial (28.5%), Latino/Hispanic (11.5%), American Indian/Alaska Native/Native Hawaiian/Pacific Islander (4.5%), Asian (2.5%), with Black students having the lowest incidence of NSSI (2.5%). Regarding sexual orientation, prevalence of engagement in NSSI was highest for students who identified as bisexual (38.0%), followed by those who were not sure of their sexual orientation (29.5%), gay or lesbian (19%), with heterosexual students having the lowest rates of NSSI (13.5%). For gender identity, students who stated they did not know if they were transgender had the highest rates of NSSI (35.0%), followed by those who identified as transgender and outside the binary of man/woman (26.5%), transmasculine (23.5%), with transfeminine participants reporting the lowest rate of NSSI (17.1%).

#### 3.1.3. Suicide Attempt

The rate of suicide attempt was highest among youth who identified as White (42%), Multiracial (32.1%), Latino/Hispanic (16.1%), American Indian/Alaska Native/Native Hawaiian/Pacific Islander (6.3%), with both Asian (1.8%) and Black students (1.8%) having the lowest incidence of suicide attempt. In terms of sexual orientation, prevalence of suicide attempt was highest for students who identified as bisexual (32.1%), followed by those students who stated they were not sure of their sexual orientation (28.6%), gay or lesbian (23.2%), with students who identified heterosexual (16.1%) having the lowest rates (16.1%). For gender identity, students who identified as either transmasculine (27.7%) or transgender and outside the binary of man/woman (27.7%) had the highest rates of suicide attempt, followed by those who stated they did not know if they were transgender (24.1%), with transfeminine students having the lowest rates of suicide attempt (20.5%).

### 3.2. Inferential Statistics

#### 3.2.1. Depression

In examining the model for recent depression, students who identified gay or lesbian (AOR = 2.57, 95% CI [1.30, 5.08]) and bisexual (AOR = 4.21, 95% CI [2.33, 7.64]), when compared to heterosexual participants, were at elevated odds of experiencing recent depression. Age, race/ethnicity, and gender identity were not significant predictors of recent depression.

#### 3.2.2. NSSI

In examining the model for NSSI, compared to transgender youth who indicated they were heterosexual, gay and lesbian respondents (AOR = 3.76, 95% CI [1.86, 7.63]) and those who did not know their sexual orientation (AOR = 3.13, 95% CI [1.71, 5.74]) were more than three times as likely to engage in NSSI, while bisexual respondents were more than four times as likely to engage in NSSI (AOR = 4.31, 95% CI [2.35, 7.88]). Compared to students who indicated they did not know if they were transgender, students who identified as transgender but not as transmasculine or transfeminine (AOR = 2.61, 95% CI [1.40, 4.85]) were at elevated odds of engagement in NSSI.

#### 3.2.3. Suicide Attempt

In examining the model for suicide attempt, compared to White respondents, American Indian/Alaska Native/Native Hawaiian/Pacific Islander respondents had 3.7 times greater odds of attempting suicide (AOR = 3.71, 95% CI [1.14, 12.07]). Compared to transgender youth who indicated they were heterosexual, lesbian and gay respondents (AOR = 2.80, 95% CI [1.32, 5.94]), bisexual respondents (AOR = 2.20, 95% CI [1.10, 4.41]), and students who indicated they did not know their sexual orientation (AOR = 2.43, 95% CI [1.14, 4.71]) were over two times as likely to report suicide attempt. Transgender youth were at an even greater odds of reporting suicide attempt: Compared to students who indicated they did not know if they were transgender or not, transfeminine students (AOR = 2.97, 95% CI [1.44, 6.10]), transmasculine students (AOR = 2.78, 95% CI [1.46, 5.29]) were almost three times as likely to report suicide attempt. Students who identified as transgender but not as transmasculine or transfeminine (AOR = 3.42, 95% CI [1.78, 6.57]) were more than three times as likely to report suicide attempt (Table 2).

## 4. Discussion and Implications

The goal of this study was to explore intra-group differences of mental health challenges amongst TGD young people. Extant research has often treated this population as a homogenous group; often, researchers compare TGD respondents either to cisgender individuals or to cisgender boys/men and cisgender girls/women, rather than examining the potential differences within this diverse population. It is crucial to better understand the unique mental health needs of this population, particularly given the high rates of mental health concerns.

The likelihood of depression was similar across TGD individuals of all genders, while different genders had differential likelihood of having experienced NSSI and suicidal attempts. This likely indicates that depression may be a more universal experience for all genders within the TGD population, partially given that this is a likely reaction to living in an inherently transphobic world. Minority stress theory has often been used to explore the impact of transphobia [50] and explain the high rates of mental health diagnoses and concerns among TGD individuals [51,52] and these findings may indicate similar experiences for the TGD youth in this study.

All sexual orientations were significant except for the likelihood of experiencing depression for those questioning their sexual orientation. This finding is not surprising, given a series of recent studies that have indicated unique experiences of TGD individuals who are also lesbian, gay, bisexual, and/or questioning their sexual orientation when it comes to mental health [38], forced sex [53], dating violence [54], school bullying [55], sexual behaviors [56], and sexual risk taking [57]. This makes clear the fact that we cannot fully separate out sexual orientation and gender from one another, while neither can we combine them together into the “LGBTQ” consolidation. Rather, we must look at the intersection of these two types of marginalization, and tailor our interventions and support here, as well as conduct more research at this intersection.

Unlike some previous studies, age was not significantly associated with any of the mental health conditions in any of the multivariate analyses. Additionally, another surprising finding was that race was only significant for American Indian/Alaska Native/Native Hawaiian/Pacific Islander TGD young people, specifically that they were almost four times more likely to attempt suicide than their White peers. This significant finding shines light on the high rates of suicidality within many of these populations [58] and that there is a need for a deeper understanding of this intersection. The lack of other significant racial findings may be because gender plays such a large role in the mental health of these young people that it negates some of the racial differences, that there are or may be forms of community support in certain BIPOC communities that mediate concerns around mental health, or that this study did not have enough racially diverse participants to statistically discover existing differences. It is also likely that these analyses did not capture potential aspects that would have exposed differences, such as everyday discrimination, interactions with police, etc. Future research must more specifically explore the intersection of race, gender, and sexual orientation to be able to best support marginalized communities.

Implications of these findings are twofold. First, there remains a need for trans-affirming mental health support for TGD young people. This need is elevated for TGD young people who are Black, Indigenous, and other people of color (BIPOC), whose needs might be better met by BIPOC therapists who can personally identify with some of their experiences. Moreover, new and existing programming to prevent and intervene in issues related to the mental health of TGD young people must consider the unique and differing needs of sub-groups within this larger population. Another way to better meet this need can be supported by more affirming local, state, and federal policies that (1) do not allow for legal discrimination or violence against this population and (2) consider the unique needs and experiences of TGD young people.

Secondly, this study sheds light on both broad and specific implications related to research. Research studies—even if not specific to TGD populations—must include TGD-inclusive variables for gender and sexual orientation. This includes allowing respondents to select multiple options and capturing gender identity (i.e., man, woman, nonbinary, agender, two spirit) separately from gender modality (i.e., cisgender, transgender). Specifically, mental and physical health studies must include these data, to provide practitioners the necessary information to treat and serve this population. Researchers must also ask more nuanced questions regarding mental health in this population, examining the specific triggers of mental health concerns (e.g., specific identities, experiences of discrimination, societal stigma). Qualitative methods such as in-depth interviews and focus groups also will allow for such nuance to be reflected in results. Such studies will allow for better understandings of what interventions can best support TGD youth navigating mental health concerns. Future research must also explore affirming interventions at the micro, mezzo, and macro levels that are created specifically for youth who are both BIPOC and TGD.

## 5. Limitations

Given that these findings are cross-sectional, there are the usual limitations on causality and incidents that would not occur within a longitudinal study. Secondly, since this study was conducted using secondary data analysis, we had no control over how questions were formulated and we had to use the framing as it was already written.

Additionally, because of the constantly evolving language around gender, some participants may have felt that the options given were not relevant to them and their identities. Ergo, they may have skipped the question or chosen an inaccurate response, potentially impacting whether they ended up in our sample of TGD young people. As a result, there may be further in-group differences we were unable to assess. We should note that Colorado, where the data were collected, is a state with specific protections for TGD individuals, especially youth. Given this, our findings may not be generalizable to TGD youth in states with fewer protections, or states with anti-TGD policies. Unfortunately, as gender identity (and sexual orientation) are frequently not collected on most state and national general surveys, additional research focusing on youth, including on topics of violence, health, school experiences, etc., should be sure to enquire about these variables. This would provide more data to facilitate a deeper exploration of TGD youths’ experiences.

## 6. Conclusions

TGD young people experience alarmingly high rates of mental health concerns, including depression, NSSI, and suicide attempts. Most of these concerns may be attributed to living in a transphobic world and the resulting discrimination, stigma, and victimization. Multiple sociodemographic characteristics were significantly related to these mental health outcomes among TGD youth. This suggests that social service providers and educators who engage with TGD young people should be acutely aware of the overall issues of mental health concerns particular to this population, and specifically those who experience multiple forms of marginalization.

## Figures and Tables

**Table 1:** Descriptive Statistics for Participants and Key Variables.

**Variables**		***N***	**%**						
Dependent variables									
Depression	No	179	45.2						
	Yes	217	54.8						
NSSI	No	196	49.5						
	Yes	200	50.5						
Suicide Attempt	No	284	71.7						
	Yes	112	28.3						
**Independent variables**				**Recent Depression**		**NSSI**		**Suicidal Attempt**	
		*N*	%	*N*	%	*N*	%	*N*	%
Race/	AI/AN/NH/PI	14	3.5	7	3.2	9	4.5	7	6.3
Ethnicity	Asian	15	3.8	5	2.3	8	4.0	2	1.8
	Black	11	2.8	3	1.4	5	2.5	2	1.8
	Latino/Hispanic	68	17.2	38	17.5	23	11.5	18	16.1
	Multiracial	96	24.2	59	27.2	57	28.5	36	32.1
	White	192	48.5	105	48.4	98	49.0	47	42.0
Sexual	Bisexual	119	30.1	86	39.6	76	38.0	36	32.1
Orientation	Gay or Lesbian	61	15.4	39	18.0	38	19.0	26	23.2
	Heterosexual	105	26.5	41	18.9	27	13.5	18	16.1
	Not Sure	111	28.0	51	23.5	59	29.5	32	28.6
Gender	Don’t Know	167	42.2	82	37.8	70	35.0	27	24.1
Identity	Transfeminine	66	16.67	37	17.1	30	15.0	23	20.5
	Transmasculine	87	21.97	49	22.6	47	23.5	31	27.68
	Trans Other	76	19.19	49	22.58	53	26.50	31	27.68

Note: AI/AN/NH/PI = American Indian or Alaska Native or Hawaiian or Other Pacific Islander.

**Table 2:** Logistic Regression Models for Predicting Mental Health among TGD (*N* = 396).

Variable	Regression 1 Recent Depression AOR [95% CI]	Regression 2 NSSI AOR [95% CI]	Regression 3 Suicide Attempt AOR [95% CI]
Age	0.88 [0.75, 1.04]	0.88 [0.74, 1.04]	1.10 [0.92, 1.32]
Race *(White)*			
AI/AN/NH/PI	0.93 [0.29, 2.92]	2.21 [0.68, 7.24]	3.71 [1.14, 12.07] *
Asian	0.37 [0.12, 1.20]	1.20 [0.39, 3.74]	0.38 [0.08, 1.85]
Black	0.36 [0.09, 1.48]	0.92 [0.25, 3.34]	0.59 [0.12, 3.05]
Latino/Hispanic	1.25 [0.69, 2.28]	0.63 [0.34, 1.17]	1.36 [0.69, 2.68]
Multiracial	1.24 [0.73, 2.12]	1.29 [0.75, 2.22]	1.44 [0.82, 2.51]
Sexual			
Orientation *(heterosexual)*			
Gay or lesbian			
Bisexual	2.57 [1.30, 5.08] **	3.76 [1.86, 7.63] ***	2.80 [1.32, 5.94] **
Not sure	4.21 [2.33, 7.64] ***	4.31 [2.35, 7.88] ***	2.20 [1.10, 4.41] *
Gender Identity *(don’t know)*	1.55 [0.87, 2.76]	3.13 [1.71, 5.74] ***	2.32 [1.14, 4.71] *
Sexual			
Transfeminine	1.64 [0.86, 3.12]	1.34 [0.70, 2.57]	2.97 [1.44, 6.10] **
Transmasculine	1.41 [0.79, 2.49]	1.71 [0.96, 3.05]	2.78 [1.46, 5.29] **
Trans other	1.30 [0.75, 2.53]	2.61 [1.40, 4.85] **	3.42 [1.78, 6.57] ***

Note: Odds ratios are adjusted for the other predictors in the model. * *p* < 0.05, ** *p* < 0.01, *** *p* < 0.001, AI/AN/NH/PI = American Indian or Alaska Native or Native Hawaiian or Other Pacific Islander.
